# Pancreaticoduodenectomy after Laparoscopic Sleeve Gastrectomy with Preservation of Gastric Arterial Inflow: A Case Report

**DOI:** 10.70352/scrj.cr.26-0456

**Published:** 2026-10-16

**Authors:** Yuta Yoshioka, Akihito Kozuki, Makoto Shinzeki, Koichiro Abe, Takumi Omine, Naoto Tsuyu, Keisuke Takahashi, Taishi Nishikawa, Hiroko Tokikuni, Kunihiko Kaneda

**Affiliations:** Department of Surgery, Kakogawa Central City Hospital, 439 Honmachi, Kakogawa, Hyogo, Japan

**Keywords:** pancreaticoduodenectomy, laparoscopic sleeve gastrectomy, pancreatic head cancer, morbid obesity, bariatric surgery, gastric arterial inflow, vascular preservation

## Abstract

**INTRODUCTION:**

Obesity is an established risk factor for pancreatic ductal adenocarcinoma. As laparoscopic sleeve gastrectomy (LSG) has become increasingly prevalent, pancreatic surgeons may encounter malignancies in patients with a surgically altered gastric anatomy. Pancreaticoduodenectomy (PD) after an LSG is rare and requires meticulous preoperative planning to prevent ischemic and anastomotic complications.

**CASE PRESENTATION:**

A 47-year-old man with morbid obesity (BMI 59.1 kg/m^2^) underwent LSG. Twenty-three days later, obstructive jaundice led to the diagnosis of pancreatic head adenocarcinoma. After neoadjuvant chemotherapy and substantial weight reduction (BMI 34.9 kg/m^2^), subtotal stomach-preserving pancreaticoduodenectomy with wedge resection of the superior mesenteric vein was performed. Preoperative CT confirmed the preservation and patency of both the left and right gastric arteries. The left gastric artery was preserved intraoperatively. Gastrojejunostomy was performed using a hand-sewn anastomosis between the greater curvature side of the sleeve stomach and the jejunum. The patient developed a grade B postoperative pancreatic fistula but was discharged with no ischemic complications.

**CONCLUSIONS:**

PD after an LSG is feasible, but careful vascular assessment, a preplanned reconstruction strategy, consideration of the patient’s metabolic and nutritional status, and careful intraoperative assessment of the remnant stomach may enhance surgical safety.

## Abbreviations

ERBD
: endoscopic retrograde biliary drainage
ERCP
: endoscopic retrograde cholangiopancreatography
EUS
: endoscopic US
ICG
: indocyanine green
LGA
: left gastric artery
LSG
: laparoscopic sleeve gastrectomy
NAC
: neoadjuvant chemotherapy
PD
: pancreaticoduodenectomy
RGA
: right gastric artery
RYGB
: Roux-en-Y gastric bypass
SMV
: superior mesenteric vein
SSPPD
: subtotal stomach-preserving pancreaticoduodenectomy

## INTRODUCTION

Obesity and diabetes mellitus are established risk factors for pancreatic ductal adenocarcinoma.^1,2)^ Epidemiological studies have demonstrated a dose–response relationship between the BMI and the risk of pancreatic cancer.^3)^ With the global increase in bariatric surgical procedures, particularly LSG, pancreatic surgeons are increasingly likely to encounter pancreatic malignancies in patients with a surgically altered upper gastrointestinal anatomy.^4–6)^

LSG removes the greater curvature and divides the short gastric vessels, resulting in a sleeve-shaped gastric remnant that is predominantly supplied by the LGA. During PD, the right gastric and right gastroepiploic vessels are typically divided. In patients undergoing LSG, an unrecognized compromise in the gastric arterial inflow may result in ischemic complications. Therefore, PD after an LSG requires individualized vascular planning.

Here, we report a case of pancreatic head cancer diagnosed shortly after an LSG in a patient with morbid obesity. We describe a structured perioperative strategy applied in this patient, emphasizing vascular assessment, consideration of metabolic and nutritional status, and avoidance of complications.

## CASE PRESENTATION

A 47-year-old man with morbid obesity (height, 167.1 cm; body weight, 164.7 kg; corresponding to a BMI of 59.1 kg/m^2^) was referred to our hospital for metabolic surgery. His medical history included type 2 diabetes mellitus treated with oral diabetes drugs, sleep apnea syndrome requiring continuous positive airway pressure, nonalcoholic fatty liver disease, dyslipidemia, hyperuricemia, and hypertension. No family history of pancreatic malignancies was reported. As part of our institutional preoperative protocol for LSG, non-contrast abdominal CT was routinely performed, whereas contrast-enhanced CT was not routinely indicated unless abnormalities were identified on non-contrast CT or during clinical evaluation. Abdominal ultrasonography was not performed before LSG.

Preoperative non-contrast CT demonstrated fatty liver and a simple hepatic cyst in segment 7. No obvious pancreatic mass, biliary dilatation, enlarged lymph nodes, or other abnormal intra-abdominal findings were identified. Preoperative laboratory findings were also unremarkable, including aspartate aminotransferase 17 U/L, alanine aminotransferase 20 U/L, alkaline phosphatase 57 U/L, γ-glutamyl transpeptidase 34 U/L, amylase 90 U/L, total bilirubin 0.73 mg/dL, and HbA1c 5.6%. Because no imaging or laboratory findings suggested pancreaticobiliary disease, serum tumor markers, including carbohydrate antigen 19-9 and carcinoembryonic antigen, were not measured before LSG. Based on these findings, no contraindication to bariatric surgery was identified, and the patient proceeded with LSG.

The LSG was performed in a standard manner. Mobilization of the greater curvature was initiated approximately 4 cm proximal to the pylorus, with division of the short gastric vessels and the left gastroepiploic artery. A tubular gastric remnant was created using a 36-Fr calibration bougie (**Fig. 1**). Surgery was completed without intraoperative complications, and the patient was discharged on POD 3.

**Fig. 1:**
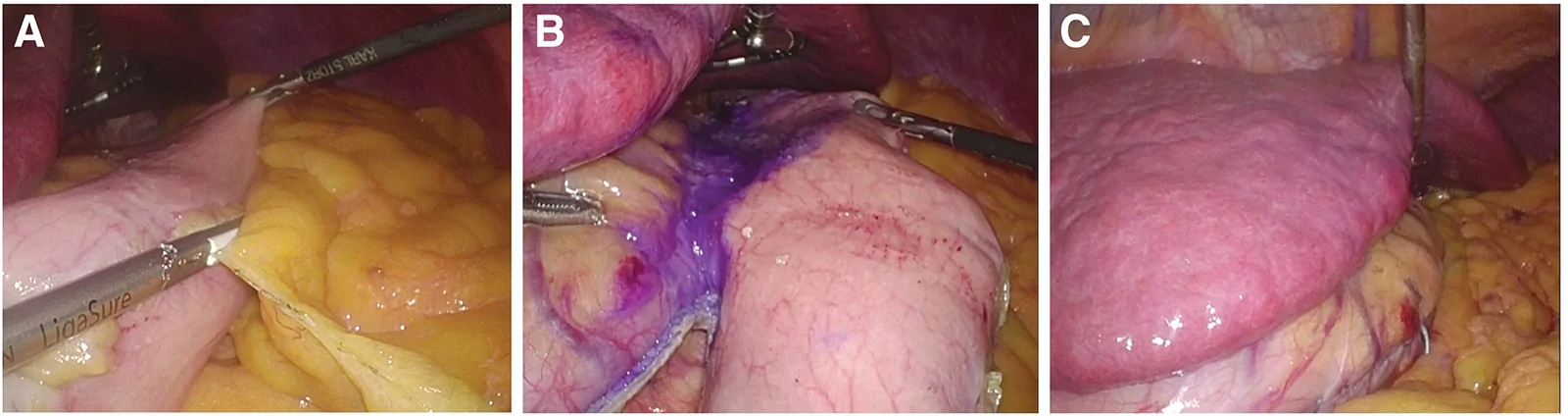
Intraoperative findings during LSG. (**A**) Mobilization of the greater curvature with division of the greater omentum along the gastric wall. (**B**) Gastric transection using a linear stapler to create a sleeve-shaped stomach. (**C**) Final appearance of the gastric sleeve on completion of the procedure. LSG, laparoscopic sleeve gastrectomy

On POD 23, routine laboratory examination revealed cholestatic liver dysfunction, with a total bilirubin level of 8.95 mg/dL. The patient presented with brown urine but denied having abdominal pain or fever.

Contrast-enhanced multidetector CT revealed a 2-cm hypovascular lesion with delayed enhancement of the pancreatic head (**Fig. 2A**, **2B**). The tumor caused distal common bile duct obstruction with upstream dilation of both the bile and pancreatic ducts. Venous-phase imaging demonstrated focal contact between the tumor and the dorsal aspect of the SMV over a short segment with no circumferential involvement or significant narrowing. Arterial encasement of the superior mesenteric artery or common hepatic artery was not observed and no distant metastases were detected. According to the Japanese Classification of Pancreatic Carcinoma, the tumor was classified as resectable (R). The tumor contact with the SMV measured approximately 43°, extending over 12.55 mm, with only 4.2% luminal narrowing. No arterial contact was identified.

**Fig. 2:**
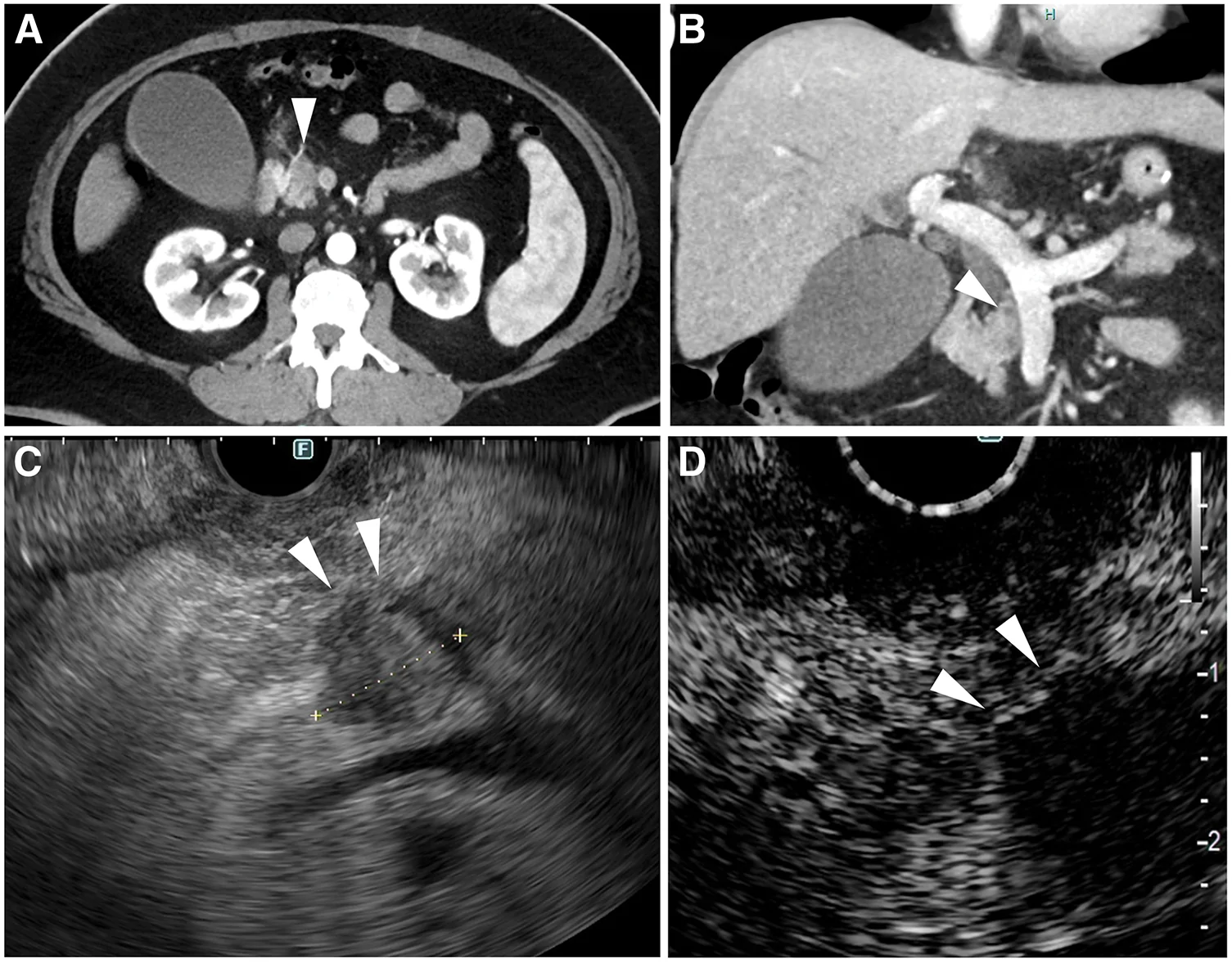
Preoperative imaging findings of pancreatic head cancer. The tumor was in contact with the dorsal aspect of the SMV without arterial encasement or invasion of the surrounding organs. (**A**) Axial view. (**B**) Coronal view. (**C**, **D**) Endoscopic ultrasonography showing an ill-defined hypoechoic mass in the pancreatic head with dilation of the distal pancreatic duct. (**D**) Contrast-enhanced endoscopic ultrasonography using Sonazoid demonstrating a hypovascular tumor. EUS-guided fine-needle biopsy confirmed the diagnosis of adenocarcinoma. SMV, superior mesenteric vein

MRI confirmed a 2-cm lesion with low signal intensity on T1-weighted imaging and diffusion restriction. Endoscopic ultrasonography (EUS) revealed an irregular hypoechoic mass in the pancreatic head with dilation of the distal pancreatic duct (**Fig. 2C**, **2D**). EUS-guided fine-needle biopsy confirmed the diagnosis of moderately differentiated pancreatic ductal adenocarcinoma. ERCP demonstrated dilation of the common bile duct and distal bile duct stenosis. Endoscopic biliary drainage was achieved by placing an ERBD tube (**Fig. 3**).

**Fig. 3:**
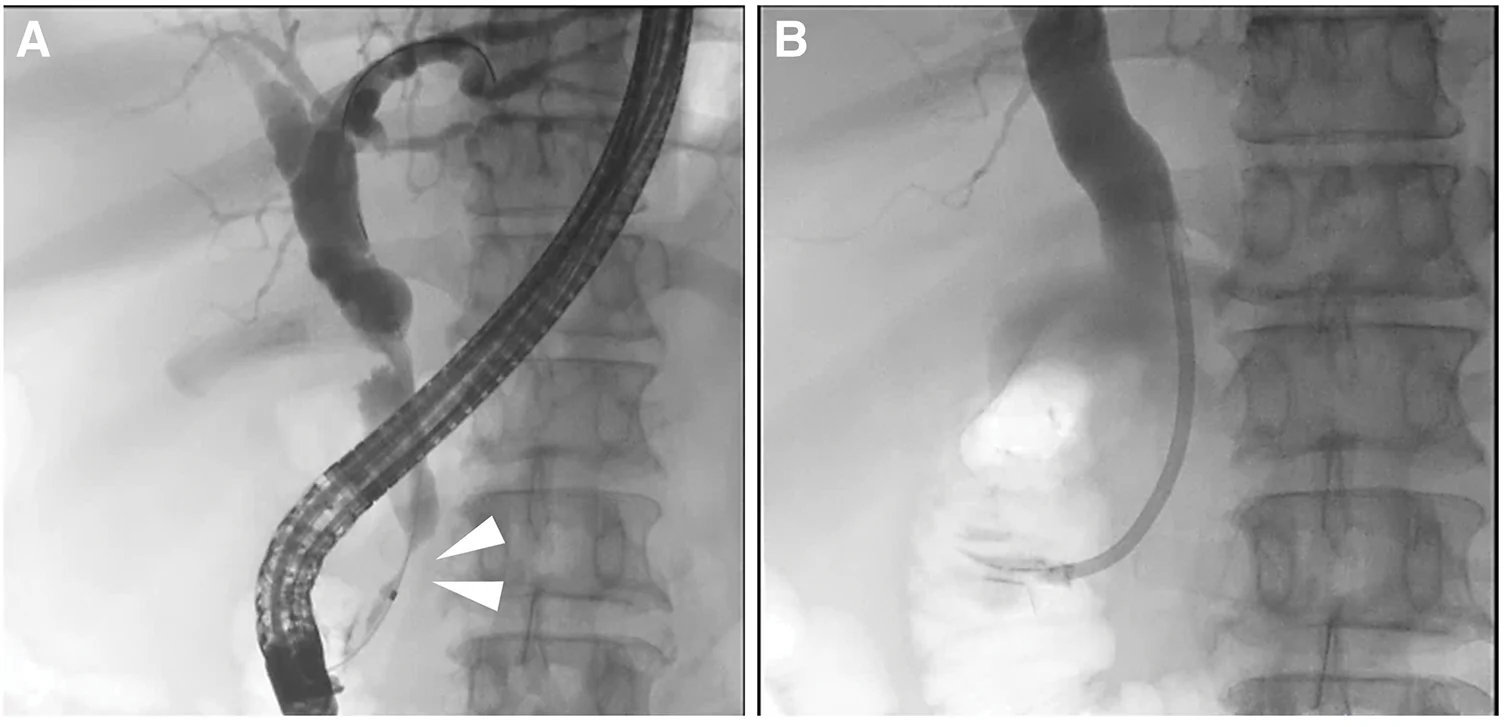
ERCP findings. (**A**) ERCP showing dilation of the common bile duct with stenosis of the distal bile duct. (**B**) ERBD tube placement performed for decompression of obstructive jaundice. ERBD, endoscopic retrograde biliary drainage; ERCP, endoscopic retrograde cholangiopancreatography

Following successful endoscopic biliary drainage and an improvement in the direct bilirubin level to 0.33 mg/dL, the patient underwent 2 planned cycles of NAC consisting of gemcitabine and S-1. The radiological response was classified as stable disease according to RECIST version 1.1. Serum CA19-9 decreased from 58.5 U/mL before NAC to 43.4 U/mL after treatment. During the first cycle, gemcitabine on day 15 was omitted because of CTCAE grade 3 neutropenia. Nevertheless, the patient completed the planned neoadjuvant treatment course and subsequently underwent PD. PET-CT scans after NAC showed no change in tumor size, but the standardized uptake value maximum (SUVmax) decreased from 7.7 to 6.0. No additional imaging findings suggestive of disease progression or distant metastases were observed.

Through dietary modification and the metabolic effects of prior LSG, the BMI decreased from 59.1 to 34.9 kg/m^2^ before surgery. Glycemic control and cardiopulmonary risk factors also improved. Changes in BMI, nutritional status, glycemic control, and CT-based skeletal muscle index during the perioperative course are summarized in **Table 1**.

**Table 1:** Perioperative changes in metabolic and nutritional parameters

Parameter	Before LSG	Before PD	6 months after PD	1 year after PD
BMI (kg/m^2^)	59.1	34.9	25.6	26.2
Albumin (g/dL)	3.7	3.3	3.1	3.4
HbA1c (%)	5.6	4.2	4.0	4.4
CT-based L3 SMI (cm^2^/m^2^)	64.55	53.24	–	60.67

Serial changes in BMI, serum albumin, HbA1c, and CT-based SMI before LSG, before PD, and during postoperative follow-up. SMI was calculated at the L3 vertebral level.

LSG, laparoscopic sleeve gastrectomy; PD, pancreaticoduodenectomy; SMI, skeletal muscle index

Because prior LSG alters the gastric vascular supply, a meticulous preoperative vascular evaluation was performed. 3D CT angiographic reconstruction confirmed the preservation and patency of both the LGA and RGA (**Fig. 4A**). No anatomical variants of the celiac trunk or the hepatic arteries were identified. Given that perfusion of the remnant sleeve stomach predominantly depends on the LGA after the division of the short gastric vessels, preservation of the LGA was defined preoperatively as essential for maintaining gastric blood flow.

**Fig. 4:**
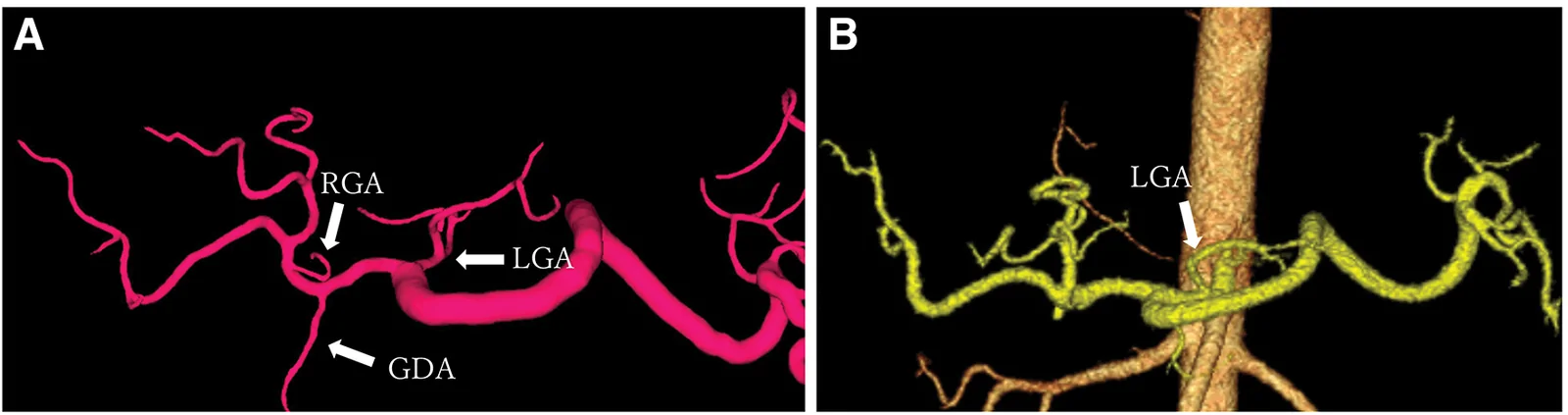
3D CT angiographic reconstruction of the gastric arterial supply. (**A**) Preoperative 3D CTA demonstrating the course of the LGA and RGA with no vascular anomalies. (**B**) POD 7 3D CT angiography confirming preservation of the LGA with no evidence of pseudoaneurysm. LGA, left gastric artery; RGA, right gastric artery

An open SSPPD was performed. No peritoneal dissemination or hepatic metastasis was observed intraoperatively. The pancreatic head tumor corresponded to the preoperative imaging findings. As adhesion between the tumor and a portion of the SMV was confirmed, wedge resection of the involved SMV was performed, followed by vascular reconstruction using continuous suturing with 5-0 polypropylene.

The LGA was carefully preserved during lymph node dissection and gastric mobilization. The RGA was divided at the time of gastric transection. Prior to the reconstruction, no apparent color changes in the gastric wall or other findings suggestive of ischemia were observed.

Reconstruction was performed through an antecolic route using a 40-cm jejunal limb and consisted of duct-to-mucosa pancreatojejunostomy, end-to-side hepaticojejunostomy, and hand-sewn gastrojejunostomy (**Fig. 5**). A Braun enteroenterostomy was additionally created. Gastrojejunostomy was performed on the greater curvature side of the sleeved stomach using a 2-layer technique, with absorbable sutures for the inner layer and interrupted seromuscular sutures for the outer layer. Particular attention was paid to mesenteric orientation to prevent torsion and ensure a tension-free anastomosis.

**Fig. 5:**
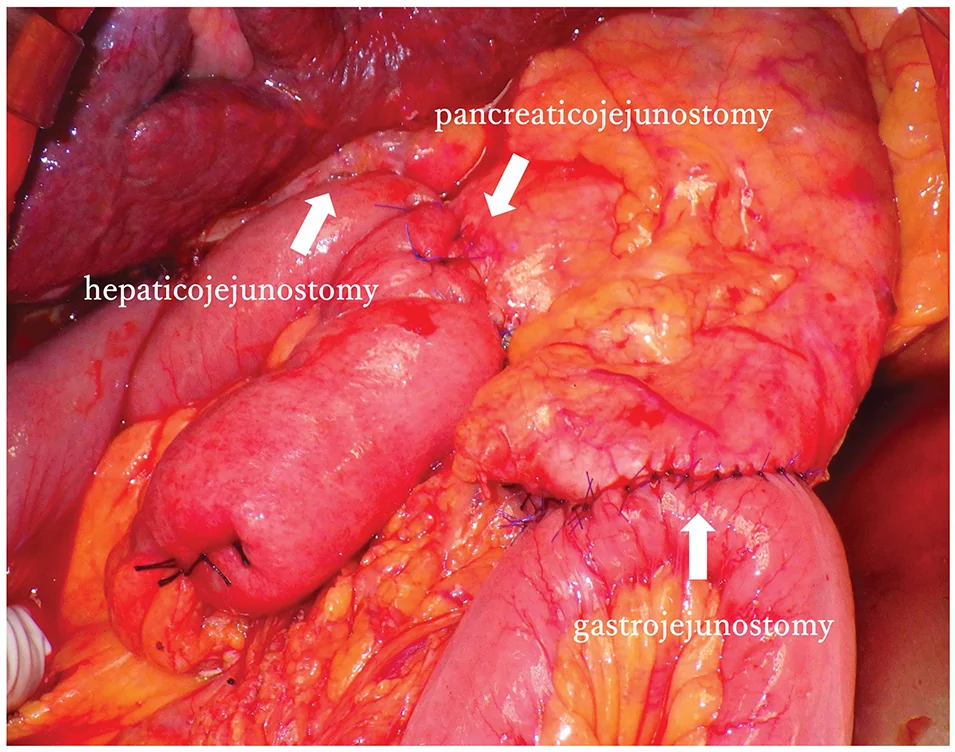
Intraoperative findings after reconstruction. Intraoperative image showing antecolic reconstruction using a 40-cm jejunal limb with pancreatojejunostomy, hepaticojejunostomy, gastrojejunostomy, and Braun enteroenterostomy.

The operative time was 8 h 14 min, and the estimated blood loss was 825 mL. No intraoperative complications were observed.

Histopathological examination revealed a moderately differentiated pancreatic ductal adenocarcinoma, classified as ypT3N1bM0, ypStage IIB. All surgical margins were negative. A total of 21 lymph nodes were retrieved, of which 4 contained metastatic disease. No lymphatic invasion was identified, whereas venous invasion and perineural invasion were present. The histopathological response to NAC was grade 1a according to the Japan Pancreas Society classification.

The postoperative course was complicated by a grade B postoperative pancreatic fistula, according to the definition of the International Study Group on Pancreatic Surgery. Although prolonged drainage was required, neither interventional radiological procedures nor reoperations were necessary. In addition, a 3D CT angiographic reconstruction performed on POD 7 demonstrated no evidence of pseudoaneurysm and confirmed preservation of the LGA (**Fig. 4B**). No evidence of remnant gastric ischemia, anastomotic leakage, or delayed gastric emptying was observed. Oral intake was gradually resumed, and the patient was discharged in a stable condition on POD 24. Long-term metabolic and nutritional parameters remained stable during follow-up (**Table 1**). No clinical evidence of severe malnutrition or impaired oral intake was observed. Adjuvant chemotherapy with S-1 was initiated after recovery from surgery and completed as planned over 6 months. Liver metastases were detected 10 months after PD. Systemic chemotherapy with gemcitabine plus nab-paclitaxel (GnP) was subsequently initiated, and the patient remained alive while receiving GnP therapy at the latest follow-up, 12 months after surgery.

## DISCUSSION

PD performed after an LSG represents an uncommon yet increasingly encountered clinical scenario in modern pancreatic surgery.^4–6)^ With the global expansion of bariatric procedures, pancreatic surgeons are increasingly confronted with malignancies arising in patients with a surgically altered anatomy. This case highlights the importance of careful vascular assessment and surgical planning before performing PD in patients with prior LSG. However, only a few studies have described PD after bariatric surgery. Previous studies have highlighted the technical challenges associated with the altered gastrointestinal anatomy and modified vascular supply following bariatric procedures.^4,5)^ After an LSG, division of the short gastric and left gastroepiploic vessels renders the remnant stomach largely dependent on the LGA. Thus, preservation of gastric arterial inflow is a critical consideration during pancreatic resection. In previously reported cases, different reconstruction strategies were adopted to maintain adequate gastric blood supply.^5)^ In the present case, preoperative 3D CTA allowed precise evaluation of the gastric arterial anatomy and facilitated a surgical strategy focused on preservation of the LGA and careful reconstruction planning. Although pancreatic cancer was diagnosed only 23 days after an LSG, suggesting that the tumor was likely present at the time of bariatric surgery, the routine preoperative assessment revealed no findings suspicious for pancreatic malignancy. Specifically, non-contrast CT showed neither a pancreatic mass nor biliary dilatation, and liver function tests, serum bilirubin, and amylase levels were all within normal limits. Therefore, there was no clinical indication for additional contrast-enhanced CT or measurement of tumor markers before LSG. Furthermore, a retrospective review of the preoperative non-contrast CT also failed to demonstrate any obvious pancreatic mass or indirect findings suggestive of pancreatic cancer. These findings suggest that the tumor was radiologically occult on routine preoperative non-contrast CT, emphasizing the diagnostic challenge of detecting occult pancreatic cancer during standard preoperative assessment for bariatric surgery. If pancreatic cancer had been diagnosed before LSG, the treatment strategy would have required careful multidisciplinary discussion, with priority given to oncological management while considering the potential impact of severe obesity on surgical safety. Incidental findings, including potentially malignant lesions, have been reported during preoperative imaging of patients undergoing bariatric surgery.^6)^ Furthermore, obesity is associated with an increased risk of pancreatic cancer and several other malignancies.^7,8)^ Therefore, the possibility of an underlying malignancy should not be overlooked in candidates for bariatric surgery, as its detection may substantially alter the therapeutic strategy and warrants careful preoperative assessment. Accordingly, we consider the principal value of this case not to be whether the pancreatic cancer was present before LSG, but rather the surgical strategy for safely performing PD after an LSG through meticulous vascular assessment, preservation of the LGA, and individualized reconstruction planning. Obesity is a well-recognized and independent risk factor for pancreatic ductal adenocarcinoma, and epidemiological studies have demonstrated a consistent dose–response relationship between increased BMI and pancreatic cancer incidence.^3)^ Obesity promotes pancreatic carcinogenesis through coordinated metabolic and inflammatory derangements, including chronic low-grade inflammation, insulin resistance with resultant hyperinsulinemia, activation of insulin-like growth factor-dependent signaling pathways, adipokine imbalance, and remodeling of the immune microenvironment.^2)^ However, visceral adiposity creates a pro-tumorigenic microenvironment that may promote tumor initiation and progression.^9)^ The occurrence of pancreatic cancer in the present patient, who initially had a BMI of 59.1 kg/m^2^, is consistent with this biological framework. The early detection of malignancy following an LSG indicates that although bariatric surgery provides significant metabolic improvement, oncogenic processes may have been established before the surgical intervention. As the global adoption of metabolic surgery continues to increase, pancreatic surgeons should be aware of the oncological burden associated with severe obesity.

In addition to these oncological considerations, LSG introduces critical anatomical alterations that directly influence subsequent PD outcomes. Resection of the greater curvature of the stomach with division of the short gastric and left gastroepiploic vessels results in a tubular gastric remnant whose perfusion largely depends on the LGA.^10,11)^ In conventional PD, the division of the right gastric and gastroepiploic vessels is the standard practice, and gastric perfusion is rarely a limiting factor in operative planning.^12,13)^ However, after an LSG, the vascular redundancy of the stomach is substantially reduced, rendering the remnant stomach predominantly dependent on inflow from the LGA to maintain perfusion. Under these circumstances, even a minor compromise in the LGA may result in profound perfusion insufficiency and potentially catastrophic ischemia of the gastric remnants. Thus, a PD following an LSG should not be approached as a routine procedure, but rather requires an individualized vascular strategy based on meticulous preoperative imaging assessment.

In this case, contrast-enhanced CT with 3D vascular reconstruction confirmed the patency of both the LGA and RGA. This information is pivotal for shaping the operative strategy. Therefore, preservation of the LGA was established as a key preoperative objective, guiding both the dissection strategy and reconstruction planning. If preservation of the LGA had not been feasible because of tumor involvement or an inadvertent intraoperative injury, arterial reconstruction would have been considered to maintain gastric arterial inflow. At our institution, this would have been performed in collaboration with cardiovascular surgeons using an end-to-end arterial anastomosis whenever technically feasible. Total gastrectomy would also have been considered as a salvage option; however, because of its greater surgical invasiveness and the increased risk of postoperative nutritional impairment in a patient who had previously undergone LSG, every effort was made to preserve the LGA. This contingency planning formed an important component of our preoperative surgical strategy. Despite the limited histopathological response to NAC (grade 1a according to the Japanese Classification of Pancreatic Carcinoma), margin-negative resection was successfully achieved. This suggests that careful vascular planning to preserve gastric arterial inflow can be accomplished without compromising oncological resection in appropriately selected patients. Most reported cases and case series of PD after bariatric surgery have involved patients with previous RYGB.^14–19)^ In contrast, PD after an LSG has rarely been reported. Küper et al.^4)^ previously described pylorus-preserving PD after an LSG for pancreatic cancer. Reported reconstruction strategies after bariatric surgery vary according to the preceding procedure and include preservation or resection of the gastric remnant, use of the pre-existing biliopancreatic limb, creation of a new pancreatobiliary limb, venting gastrojejunostomy, and omega reconstruction with Braun jejunojejunostomy (**Table 2**).^4,14–19)^ After an LSG, preservation of arterial inflow to the tubular gastric remnant is a particular concern because the short gastric and left gastroepiploic vessels have already been divided. To our knowledge, detailed reports combining preoperative 3D assessment of the gastric arterial anatomy, deliberate preservation of the LGA, stomach-preserving reconstruction, and longitudinal nutritional evaluation remain limited. These features represent the principal novelty of the present case. These findings underscore the importance of meticulous preoperative vascular mapping and individualized reconstruction planning in patients with surgically altered gastric anatomy.^12)^

**Table 2:** Comparison of reported cases of PD after bariatric surgery

Year	Author	Previous bariatric surgery	Gastric remnant	Gastric arterial management	Reconstruction
2009	Küper et al.^4)^	LSG	Preserved	Not clearly reported	Pylorus-preserving PD; detailed reconstruction not clearly reported
2022	Mak et al.^14)^	RYGB	Preserved	Not reported	Distal segment of the original biliopancreatic limb used for venting gastrojejunostomy; new jejunal limb used for PJ/HJ + new JJ
2023	Jackson et al.^15)^	RYGB	Preserved	Not reported	PJ and HJ reconstructed using the existing RYGB biliopancreatic limb; gastric remnant retained
2023	Rojas et al.^16)^	RYGB	Not clearly reported	Not reported	Robotic PD; detailed reconstruction not clearly reported
2023	Yaqub et al.^17)^	RYGB	Resected in all 6 patients	Not reported	Pre-existing biliopancreatic limb used for PJ/HJ in most patients
2024	Orsi et al.^18)^	RYGB	Preserved	Not reported	Old common channel used for PJ, HJ, and remnant gastrojejunostomy
2025	Silviu-Tiberiu et al.^19)^	RYGB	Preserved	Not reported	Laparoscopic omega-type reconstruction with Braun jejunojejunostomy
Present case	Yoshioka et al.	LSG	Preserved	Preoperative 3D assessment; LGA preserved	Antecolic reconstruction + Braun enteroenterostomy

Comparison of the present case with previously reported cases of PD after bariatric surgery, focusing on the type of previous bariatric procedure, management of the gastric remnant and gastric arterial inflow, and reconstruction strategy.

LSG, laparoscopic sleeve gastrectomy; PD, pancreaticoduodenectomy; RYGB, Roux-en-Y gastric bypass

An additional surgical consideration in this setting is whether gastric resection or total gastrectomy should be performed during PD following an LSG. If adequate gastric arterial inflow can be safely preserved, maintaining the remnant stomach may help avoid the nutritional disadvantages associated with total gastrectomy. Consistent with this concept, our patient maintained satisfactory long-term nutritional status after surgery. Serum albumin remained stable, glycemic control was preserved, and the skeletal muscle index recovered during follow-up, without evidence of severe malnutrition. These findings support the feasibility of remnant stomach preservation after PD in selected patients following an LSG. In the present case, preoperative vascular evaluation confirmed an intact gastric arterial supply and preservation of the LGA allowed for safe reconstruction without the need for additional gastric resection.

Gastric perfusion assessment is an important consideration when performing PD following sleeve gastrectomy. Although the preservation of macroscopic arterial inflow is essential, it does not necessarily ensure adequate microvascular perfusion. ICG fluorescence imaging has recently been introduced as a useful modality for the real-time evaluation of tissue perfusion during hepatobiliary and pancreatic surgery.^20,21)^ In the present case, gastric perfusion was assessed on the basis of careful preservation of the LGA and intraoperative visual assessment of the remnant stomach, including its color and arterial pulsation. Because the LGA was clearly preserved, arterial pulsation was readily palpable, and the remnant stomach showed no discoloration suggestive of ischemia, additional intraoperative perfusion imaging was not considered necessary. However, we acknowledge that visual inspection and palpation are inherently subjective and cannot completely evaluate microvascular perfusion of the gastric wall and gastrojejunostomy. When arterial pulsation cannot be confidently confirmed or the appearance of the remnant stomach is equivocal, macroscopic assessment alone may be insufficient. In such situations, intraoperative ICG fluorescence imaging should be considered to provide objective confirmation of gastric perfusion. Although ICG fluorescence imaging was not used in the present case, it may be a valuable adjunct for objective assessment of gastric perfusion when available, particularly when macroscopic assessment is inconclusive.

The influence of obesity on postoperative morbidity after PD provides an important context for this case report. Previous studies have consistently demonstrated that obesity is associated with increased rates of postoperative pancreatic fistulae, wound complications, and prolonged operative time.^22,23)^ Excess visceral adiposity increases operative complexity and may impair anastomotic healing through fatty pancreatic infiltration and an enhanced inflammatory response.^22)^ In the present patient, BMI decreased markedly from 59.1 to 34.9 kg/m^2^ before resection. Substantial weight reduction before PD improved operative exposure. In addition, glycemic control improved, and serum albumin remained relatively preserved, despite a reduction in the skeletal muscle mass. Although these metabolic changes may have contributed to favorable operative conditions, their direct impact on perioperative outcomes cannot be determined from this single case. As summarized in **Table 1**, glycemic control improved (HbA1c 5.6% to 4.2%) and serum albumin remained relatively preserved (3.7 to 3.3 g/dL), despite a reduction in the CT-based skeletal muscle index (64.55 to 53.24 cm^2^/m^2^).

Although a grade B postoperative pancreatic fistula developed according to the criteria of the International Study Group on Pancreatic Surgery,^24)^ it was successfully managed conservatively without progression to major morbidities. Importantly, no gastric ischemia or functional gastric complications were observed postoperatively. From a broader perspective, PD after an LSG should be recognized as a technically unique operative scenario requiring individualized vascular planning.^25)^ Safe surgical management may benefit from integrating meticulous preoperative vascular mapping, consideration of metabolic and nutritional status, deliberate reconstruction planning, and careful intraoperative assessment of gastric arterial inflow and the remnant stomach. Collectively, these principles may provide a practical framework for safely performing pancreatic resection in patients who have previously undergone bariatric surgery.

This study has several limitations that warrant consideration. First, this report described a single case; therefore, the generalizability of the proposed surgical strategy remains unclear. Second, although careful preservation of the LGA and intraoperative visual assessment suggested adequate perfusion of the remnant stomach, an objective intraoperative perfusion assessment was not performed. Third, although serial assessments demonstrated improved glycemic control and relatively preserved nutritional status despite a reduction in skeletal muscle mass, the extent to which these metabolic changes contributed to the favorable perioperative outcomes cannot be determined from this single case. Further studies with larger patient cohorts are warranted to validate these findings.

Despite these limitations, this case report provides practical insights into the surgical management of pancreatic head cancer after an LSG in a patient with morbid obesity. This highlights that successful PD in this setting depends not only on technical expertise but also on meticulous preoperative planning based on a thorough understanding of the altered vascular anatomy.

## CONCLUSIONS

As bariatric surgery has become increasingly prevalent worldwide, careful vascular assessment and preservation of the gastric arterial inflow should be considered when performing PD after an LSG.

## DECLARATIONS

### Availability of data and materials

Not applicable. Data cannot be shared due to patient privacy concerns.
